# Enrichment and Reduction of Microsatellite Regions in the Myxoma Virus Genome Following Species Jump to the Iberian Hare (*Lepus granatensis*)

**DOI:** 10.1155/tbed/3847131

**Published:** 2026-04-18

**Authors:** A. Menéndez-Manjón, B. Cardoso, S. Castro-Scholten, M. Agüero, M. Duran, D. Buitrago, A. M. Lopes, J. Abrantes, M. Martinez-Haro, D. Cano-Terriza, D. Jiménez-Martín, L. Camacho-Sillero, I. Nicieza, S. Arenas-Vicente, I. Calonge-Sanz, F. Parra, A. L. Alvarez, P. Domínguez, J. M. Martín-Alonso, I. García-Bocanegra, K. P. Dalton

**Affiliations:** ^1^ Instituto Universitario de Biotecnología de Asturias, Departamento de Bioquímica y Biología Molecular, Edificio Santiago Gascón, Universidad de Oviedo, Campus El Cristo, Oviedo, 33006, Spain, uniovi.es; ^2^ Grupo Sanidad y Biotecnología (SaBio), Instituto de Investigación en Recursos Cinegéticos (IREC), UCLM-CSIC-JCCM, Ciudad Real, Spain, irec.es; ^3^ Centro de Investigação em Biodiversidade e Recursos Genéticos (CIBIO), InBIO Laboratório Associado, Universidade do Porto, Vairão, Portugal, up.pt; ^4^ Departamento de Biologia, Faculdade de Ciências da Universidade do Porto, Porto, Portugal, up.pt; ^5^ BIOPOLIS Program in Genomics, Biodiversity and Land Planning, CIBIO, Vairão, Portugal; ^6^ Departamento de Sanidad Animal, Grupo de Investigación en Sanidad Animal y Zoonosis (GISAZ), UIC Zoonosis y Enfermedades Emergentes ENZOEM, Universidad de Córdoba, Córdoba, Spain, uco.es; ^7^ Laboratorio Central de Veterinaria-, Subdirección General de Laboratorios de Sanidad Animal y Vegetal, MAPA, Ctra M106, Km 1,4, 28110, Algete, Madrid, Spain; ^8^ UMIB-Unit for Multidisciplinary Research in Biomedicine, ICBAS-School of Medicine and Biomedical Sciences, University of Porto, Porto, Portugal, up.pt; ^9^ ITR, Laboratory for Integrative and Translational Research in Population Health, Porto, Portugal; ^10^ Centro de Investigación Agroambiental El Chaparrillo, Instituto Regional de Investigación y Desarrollo Agroalimentario y Forestal de Castilla La Mancha (IRIAF), Ciudad Real, Spain; ^11^ Instituto de Investigación en Recursos Cinegéticos, IREC (CSIC, UCLM, JCCM), Ronda de Toledo 12, Ciudad Real, 13005, Spain, irec.es; ^12^ CIBERINFEC, ISCIII - CIBER de Enfermedades Infecciosas, Instituto de Salud Carlos III, Madrid, Spain, isciii.es; ^13^ Programa de Vigilancia Epidemiológica Fauna Silvestre (PVE), Consejería de Agricultura, Ganadería, Pesca y Desarrollo Sostenible, Junta de Andalucía, Málaga, Spain, juntadeandalucia.es; ^14^ Hipra, Avda. la Selva, 135, Amer, Girona, 17170, Spain

**Keywords:** emergent poxvirus, Iberian hare, microsatellite evolution, myxoma virus, myxomatosis

## Abstract

Myxoma virus (MYXV), the causative agent of myxomatosis, is endemic in wild populations of the European rabbit (*Oryctolagus cuniculus*). In 2018, the virus acquired four genes (*M157L*, *M158L*, *M159L* and *M160L*) from a yet unidentified source and gained the capacity to infect the Iberian hare (*Lepus granatensis*). The new viral strain, termed ha‐MYXV (hare myxoma virus), continues to circulate within Iberian hare populations, causing a dramatic decline in numbers of this species. To analyse the genetic stability of the emergent ha‐MYXV, following its host species jump, we sequenced the novel four‐gene cassette in 106 samples collected over an 8‐year period (2018–2025) from Iberian hares and wild and domestic rabbits throughout Spain. Samples were either collected as part of an active surveillance campaign or through passive sampling of diseased animals. Our results show that M157 and M158 are completely conserved in all samples, while M160 demonstrated occasional sporadic mutations. However, gene *M159L*, the C7L‐like host range virulence factor, demonstrated simple sequence repeat (SSR) trinucleotide expansions and contractions in ~60% of samples analysed. Further analysis showed that ha‐MYXV contains unique SSR in genes associated with early stop mutations. Sequencing of the ha‐MYXV‐specific SSR regions in field samples shows a large degree of variation. These findings indicate that the variable nature of SSR in the emerging virus contrasts with the stability of the other novel genes present in the ha‐MYXV genome. This study highlights the need for continued surveillance in order to better understand virus evolution following a cross‐species virus transmission event.

## 1. Introduction

Micro‐satellites, or simple sequence repeats (SSRs), are regions of repetitive DNA sequences found in all genomes occurring in coding and non‐coding regions. They contain tandem repeats of mono‐, di‐, tri‐, tetra‐, penta‐ or hexanucleotides. The number of repeats can range from 5 to 50 times with a minimum total array size of 8 nucleotides (nts) [1]. Due to their highly polymorphic nature, such regions are extensively used for DNA profiling and can be used for the molecular characterisation and differentiation of closely related virus strains [2, 3], including poxviruses [4–7].

The myxoma virus (MYXV), formally known as Leporipoxvirus myxoma (family *Poxviridae*) [8], is the aetiological agent of myxomatosis. Myxomatosis is endemic in Spain, which is part of the native distribution range of the European rabbit (*Oryctolagus cuniculus*) [9], having a marked negative effect on wild [10–12] and domestic rabbits [13]. MYXV has a double‐stranded DNA genome of ~161 kb, containing terminal inverted repeat regions (TIR) and encoding 171 open reading frames (ORF) [14]. MYXV is considered a paradigmatic example of host–pathogen coevolution following a species jump [15, 16]. For more than 70 years following its release as a biocontrol agent in Australia and Europe, close monitoring has demonstrated the complex evolution of host and pathogen. Currently, MYXV continues to evolve and cause new disease phenotypes in rabbits [17].

In mid‐2018, a myxomatosis outbreak was detected in southern and central Spain, affecting the Iberian hare (*Lepus granatensis*) [18], a species indigenous and unique to the Iberian Peninsula. A high mortality rate (over 50%) in hare populations was detected [18] as the disease spread, reaching most of the Iberian Peninsula, where the Iberian hare is present [19], including southern Portugal [20]. The subsequent negative effect on the Iberian hare population has been linked to the emergence of myxomatosis in this species [21].

MYXV isolated from infected Iberian hare tissues was identified as a novel recombinant MYXV strain and denominated MYXV‐Tol, or hare myxoma virus (ha‐MYXV) [22, 23].

The ha‐MYXV genome showed significant differences compared with the Lausanne MYXV genome (reference strain for European MYXV Accession Number NC_001132; MYXV‐Lau). Genomic analysis revealed the presence of four additional genes designated *M157L*, *M158L*, *M159L* and *M160L*, which showed homology with the MYXV‐Lau genes *M060R* (NP_051774, similar to VACV J1R, required for DNA packaging), *M061R* (GenBank: AAF14949.1, thymidine kinase; similar to VACV J2R), *M064R* (GenBank: AAF14952, similar to VACV host range factor C7L) and *M065R* (GenBank: AAF14953, similar to VACV J3R, a poly(A) polymerase small subunit), respectively [22, 23]. This novel four‐gene cassette (~2.8 kb), termed Ins‐H1, is present within the *M009L* gene [23]. In the Ins‐H1 sequence, M159 is essential for ha‐MYXV replication in hare cells in vitro, supporting its role in determining host specificity [24]. In addition, the ORFs of the following genes were truncated in ha‐MYXV when compared to MYXV, *M009L*, *M036L* and *M152R* [22, 23], which encode a Kelch repeat protein M‐T9 (GenBank NP_051723 UniProtKB P08073), a leucine zipper motif‐containing protein (GenBank: AAF14924; homologous to VACV O1L UniProtKB P21093), and SERP3 (GenBank: AAF15039 serine proteinase inhibitors), respectively, whose putative functions are designated due to homology with other poxviruses [14]. Ha‐MXYV has been associated with high mortality on rabbit farms [25], and recent surveys have detected its presence in the European brown hare (*Lepus europaeus*) in Spain [26] and northwestern Europe [27].

As an emerging virus in a novel ecological niche, the genetic stability of ha‐MYXV is of particular interest. The objective of this study was to monitor the sequence of the ha‐MYXV‐specific Ins‐H1 region over the 8‐year period since its emergence.

## 2. Material and Methods

### 2.1. Study Design and Sample Collection

**Figure 1:**
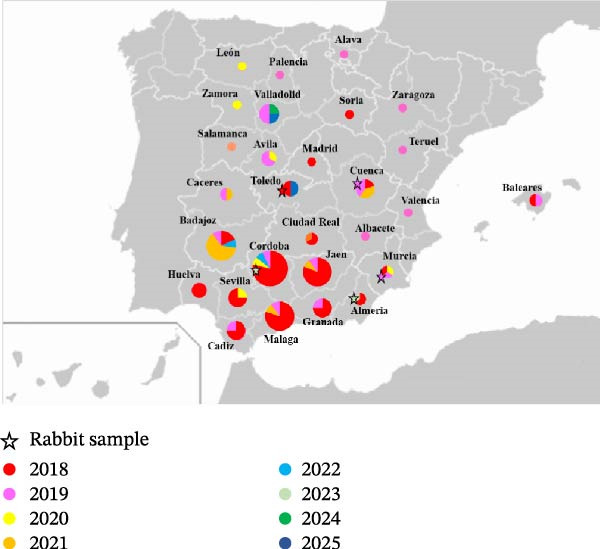
Map of Spain indicating the province of origin of ha‐MYXV‐positive samples collected between the years 2018 and 2025. The grey lines indicate the separation between provinces, and province names are shown. Data is presented as a nested bubble plot with dot and wedge sizes proportional to the number of samples collected from each province. The years of sample collection are indicated by colour; 2018 is shown in red, 2019 in pink, 2020 in yellow, 2021 in orange, 2022 in light blue, 2023 in light green, 2024 in dark green and 2025 in dark blue. Rabbit samples are indicated by the star shapes. More detailed information can be found in Supporting Information 1: Table S1.

A total of 106 lagomorph specimens were included in the present study, comprising 101 Iberian hares, four wild rabbits and one domestic rabbit from a commercial farm. Sampling was conducted through passive surveillance of individuals found dead, as well as through active surveillance of legally hunted wild lagomorphs during authorised hunting events. Specimens were collected over an 8‐year period, from 2018 to 2025, across 28 provinces in Spain (Figure 1). Following collection, the presence of MYXV DNA was analysed using conventional PCR protocols targeting two conserved genes, *M071L* and *M029L* [28].

For ha‐MYXV characterisation, each positive sample was assigned a unique identification code based on the province of collection, the year/hunting period and a registration identifier (e.g., Jaen/18‐L37 sample L37, collected in Jaen, in 2018). The full sample list is shown in Supporting Information 1: Table S1.

### 2.2. DNA Extraction

The extraction of total DNA was carried out from spleen or eyelid tissue samples using the QIAamp DNA Mini Kit following the manufacturer’s instructions (QIAGEN GmbH, Hilden, Germany). Twenty‐five mg of eyelid tissue and/or 10 mg of spleen were finely chopped and resuspended in 180 μL of lysis buffer (ATL) and 20 μL of proteinase K, then incubated overnight at 37°C. The next day, 200 μL of buffer AL was added, incubated for 10 min at 70°C, and 200 μL of 96% ethanol was added. Subsequently, the samples were applied to spin columns and centrifuged (6,000 ×*g*) for 1 min. They were then washed with AW1 buffer (6,000 ×*g*, 1 min) and AW2 buffer (20,000 ×*g*, 3 min), and finally eluted in 200 μL of nuclease‐free sterile water (6,000 ×*g*, 1 min) and stored at −20°C until use.

### 2.3. Primer Design

Due to the size of the Ins‐H1 insert of the ha‐MYXV, a series of primers was designed to amplify the four genes (*M157L*, *M158L*, *M159L* and *M160L*) as overlapping amplicons of ~500 bp. The primers were designed using Geneious Prime 2023.2 software based on the sequence of the original natural recombinant ha‐MYXV strain (MYXV strain Tol08‐18 GenBank Accession Number MK340973.1) isolated from an Iberian hare. Primers were purchased from Integrated DNA Technologies (IDT) and used from a stock solution at a concentration of 10 μM. Oligonucleotides used to sequence the *M009L*, *M036L* [29] and *M152R* [30] have been previously described. The nucleotide sequences of the primers used in the study, along with their corresponding information, are shown in Table 1.

**Table 1:** Information regarding primers used in the study for amplification and sequencing.

Primer	Sequence (5′–3′)	Orientation	Position in MK340973.1 (nt)	Reference
hMV11800	CCACGTACGTGACTCGTC	Forward	11,778–11,795	This study
MV12140	CTTCGTCTACGCCTCCTACG	Forward	12,116–12,135	26
hMV12250	CTCCGGCATTAAACTGTATTC	Forward	12,192–12,212	26
hMV12300r	CGCTATAATAAGCAGGGAGG	Reverse	12,333–12,352	This study
hMV12713	CCTCATTTCAGCAGAATAC	Forward	12713–12731	26
hMV12770	GGTGCGAATGGTTGCAAC	Forward	12,735–12,752	26
hMV12800r	GCGGCGTCTAGTTTAAAATGG	Reverse	12,812–12,832	26
hMV13290	GCACTTTCAGGTTCGTATTC	Forward	13,260–13,279	26
hMV13300r	CCAATGTCTATGGAAAAACCG	Reverse	13,313–13,333	26
hMV13419r	GAAGGTGAAGATGATGATG	Reverse	13,437–13,419	26
hMV13700	GGGTATCCATCTGTATATAAC	Forward	13,673–13,693	26
hMV13800r	CCGGATTGGAGTGAAGTG	Reverse	13,840–13,857	26
hMV14300	GATGAAGGACAGTTTTTTCCG	Reverse	14,318–14,338	26
hMV14300r	CCTTCGTTAGCCATGTTC	Forward	14,280–14,297	26
hMV14577r	CATGGCAACTTACGATGG	Reverse	14,594–14,577	26
hMV14790	GGTTGAGGCAACTACAAAATC	Forward	14,736–14,756	This study
hMV14800r	CGCTCCATTATCGGAGG	Reverse	14,873–14,889	26
hMV15295	GGACACGCGATCATATCC	Forward	15,271–15,288	This study
hMV15300r	CCGTAGACGACGTAGACG	Reverse	15,338–15,355	This study
hMV15800r	GACGTTTCTCGGGCATAAAG	Reverse	15,861–15,880	This study

### 2.4. Molecular Analysis

Amplification of the M009L gene region, which contains the recombinant insert characteristic of ha‐MYXV, was performed by conventional PCR following the protocol described by Cardoso et al. [26]. All 106 samples tested positive for ha‐MYXV infection by detection of the Ins‐H1 region, the total length of which is 2752 bp; 12,323–15,074 bp genome positions in the reference sequence isolate ha‐MYXV_L3 Toledo 8/18 (GenBank Accession MK340973). Additional PCR amplifications using the primers described in section 2.3 were carried out in a final volume of 50 μL using the LA Taq kit (Takara) following the manufacturer’s recommendations.

Amplicons were analysed on a 1% agarose gel (Agarose D1 Low EEO; Conda Laboratories) made with 1X TAE buffer and stained with RedSafe (iNtRON Biotechnology) using the O’GeneRuler 1 kb Plus DNA Ladder marker (Thermo Scientific) as a reference. Gels were visualised using a Gel Logic 212 Pro Camera (Carestream and Carestream SI ME Software).

The PCR products were cleaned up using the Wizard SV Gel and PCR Clean‐Up System (Promega), following the manufacturer’s instructions. To sequence the DNA products, up to 50 ng of each PCR product was mixed with 5 μM of the appropriate oligonucleotide, and its nucleotide sequence determined at the Sequencing Services of the University of Oviedo.

### 2.5. Bioinformatic Analysis

Sequence chromatograms were processed and analysed using ChromasLite (Technelysium Ltd, Australia) and Geneious Prime (GraphPad Software, USA) software. The manually corrected sequences were compared using BLAST to those on the NCBI database and aligned to the ha‐MYXV using Geneious Prime Software. Individual contigs were constructed from the overlapping sequence reads for each isolate. Heatmaps were generated using RStudio (Posit team, 2025. RStudio: Integrated Development Environment for R. Posit Software, PBC, Boston, MA. URL http://www.posit.co/).

Microsatellites Explorer (https://www.microsatellitesexplorer.com) is a web‐based repository that allows the identification and analysis of short tandem repeat (STR) sequences within genomes [31]. Searches were conducted on 23^rd^ March 2025, and the STR data regarding the MYXV Lausanne and ha‐MYXV L3 substrain (NCBI Genome Browsers GCF_000843685.1 and GCA_023532105.1, respectively) were downloaded and analysed in Microsoft Excel.

## 3. Results

### 3.1. Sample Analysis

Of the 106 samples analysed, 101 were obtained from Iberian hares and five from the European rabbit. Many of the samples (*n* = 77) were collected from the epicentre of the initial outbreak in 2018 (*n* = 54) and 2019 (*n* = 23) in the south central part of Spain (Figure 1). These samples were obtained through passive surveillance, and all sampled individuals were suspected of having myxomatosis due to the observation of characteristic lesions.

Thirty (30) samples were collected between 2020 and 2025, of which 15 were from active surveillance that included five animals with no symptoms of myxomatosis (Supporting Information 1: Table S1). The single province with the most samples was Córdoba, with 15 samples collected over 6 years (Figure 1). Rabbit samples were from four wild rabbits collected in Toledo in 2018, Cuenca in 2019, Cordoba in 2023 and Almeria in 2023, and one domestic rabbit from a rabbit farm in Murcia in 2019.

### 3.2. Comparison of Ins‐H1 Sequences

**Figure 2:**
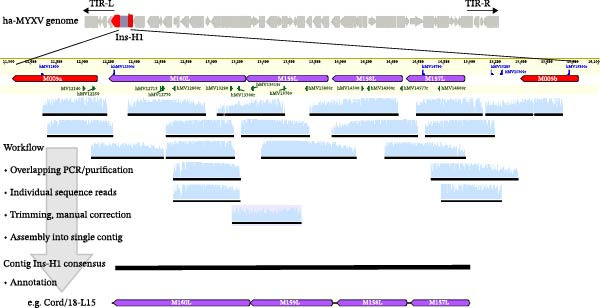
Workflow for the sequencing and contig assembly for each of the 106 ha‐MYXV isolates analysed in this study. The relative position of the Ins‐H1 region in the MYXV genome is shown. Individual sequence chromatograms (shown in light blue) are trimmed and ambiguous bases corrected when possible before assembly into a consensus contig (black line). The position and direction of primers used for amplification and sequencing are indicated by blue triangles (described in Table 1); additional primers are indicated with green triangles and have been previously published (26). The sequence was annotated, and potential ORFs identified by comparison with the reference ha‐MYXV sequence. In the example shown, 20 individual Sanger sequencing reads are used to assemble the contig for isolate Cord/18‐L15.

The region containing the four ORFs (*M157L*, *M158L*, *M159L* and *M160L*) (12,323 to 15,074 in reference strain ha‐MYXV_L3 Toledo 8/18 [GenBank Accession MK340973]), was fully sequenced in all 106 samples using a panel of 20 oligonucleotides (Table 1 and [26]). Overlapping sequence reads were used to construct contigs for each sample (Figure 2). These Ins‐H1 full‐length contig sequences were used in multiple sequence alignment analysis (data not shown).

Details of the mutations detected in each isolate are shown in Supporting Information 2: Table S2. The minimum number of nucleotide differences was three (35 isolates). These three mutations corresponded to the fixing of ambiguous nts to C at positions 12,912, 13,083 and 13,085 in gene *M160L* and were present in all strains (Supporting Information 2: Table S2). Apart from these three nucleotide differences, 35 isolates were identical to the reference strain. The maximum number of differences was 45 (isolate Vall/24‐25IT_8). This gave % identities ranging from 98.4% to 99.9% when comparing isolate sequences to MK340973. The minimum % identity was 97.7% when comparing between isolates (e.g. 64 differences between isolates Jaen/18‐L37 and Cord/23_25IT_14). A heatmap matrix of % identity of all 106 samples is shown in Supporting Information 3: Table S3. Overall similarity to the reference strain was very high (>99.1%) and stable across several years, with samples from the same province showing minimal variation. Supporting Information 4: Table S4 provides a simplified % identity matrix indicating isolate identification, province, year of sampling and % identity with regard to the reference ha‐MYXV sequence. The greatest divergence was observed in the most recent samples (2023–2025), where identity ranged from 98.4% to 99.1%. Three earlier isolates also showed slightly greater variation, one sample from 2018 (Jaen/18‐L37 98.6% identity) and two samples from 2021 (Bad/21‐ha17 and Bad/21‐ha20, 98.9% and 98.7% identity, respectively).

### 3.3. Sequence Analysis of the *M157L*, *M158L*, *M159L* and *M160L*

The sequence of each of the four ORFs present within region Ins‐H1 was studied individually. Table 2 shows a summary of the main findings.

**Table 2:** Number of isolates containing mutations in ha‐MYXV specific genes *M157L*, *M158L*, *M159L* and *M160L*.

Gene	Number of isolates with mutations	Mutation type
*M157L*	0	Not applicable
*M158L*	0	Not applicable
*M159L*	57 (Iberian hare) 3 (European rabbit)	25 isolates with triplet deletions (including three rabbit samples) 35 isolates with triplet expansions 3 isolates with combined triplet expansions and deletions
*M160L*	15 (Iberian hare) 1 (European rabbit)	8 isolates with truncations to protein • 1 isolate: Δ8‐351(end) • 1 isolate: Δ31‐351(end; including one rabbit sample • 6 isolates: Δ137‐351(end)

*M157L* and *M158L* sequences were conserved in all samples with no mutations detected in any of the 106 samples analysed. These genes are 453 nt and 546 nt, respectively, and account for a total of 36.3% of the Ins‐H1 region.

The *M160L* gene is 1056 bp in length, containing a predicted ORF of 351 amino acids and accounting for 38% of Ins‐H1. Ten synonymous or non‐synonymous mutations were detected within *M160L*. Three mutations (positions 12,662, 13,044 and 13,134) lead to the introduction of ambiguous sites (Supporting Information 2: Table S2). Three mutations occurred in the same isolate (Vall/19‐2585_2) (13,020 R‐K and 13,022 and 13,031 both silent). Six isolates (Cac/19‐2681_8; Avil/19‐2741_10; Bal/18‐3532_5; Tol/18‐1981_4/; Avil/19‐2815_1 and Pal/19‐2865_4) shared a mutation that led to the change in codon TTA‐TAA and incorporation of an early stop codon (Δ137‐351 (end), leading to a loss of 215 amino acids) (Supporting Information 2: Table S2). Additionally, two other isolates (Cord/23‐25IT_14 [collected from a rabbit] and Cord/20‐ha33) contained an insertion of a thymidine residue at position 13,296 or 13,365, respectively, which would provoke the truncation of M160 and the loss of 320 or 343 amino acids of the predicted ORF (Supporting Information 2: Table S2).

### 3.4. Triplet Expansion and Retraction in ORF *M159L*

The Ins‐H1 region of ha‐MYXV covering the four unique genes is 2752 bp in length in ha‐MYXV. However, the contig lengths for 61 samples ranged from 2711 to 2767 bp, indicating insertions of up to 15 bp and deletions up to 41 bp. These insertions and deletions were almost exclusively present in the *M159L* ORF region (except for the single base insertions/deletions previously mentioned corresponding to *M160L*). The *M159L* gene is 636 bp in length, containing a predicted ORF of 211 amino acids and accounting for 23.1% of Ins‐H1. The insertions or deletions present in ORF 159 occurred as triplets of nts, therefore maintaining the predicted ORF integrity (Table 3, Supporting Information 2: Table S2). Deletions of the triplet ATC varied from one to eight times, while ATC was inserted two to five times (Table 3, Supporting Information 2: Table S2) in the region spanning the C‐terminal tail of M159. While 46 samples showed no mutations, ATC expansions or retractions were detected in 60 isolates (56.6% of samples) and were present as 17 variant types, depending on the number and location of insertions and deletions. Samples collected following virus emergence between 2018 and 2021 (all through passive vigilance) included 35 samples with triplet insertions. From 2021 onwards, deletions in this region became predominant, as observed in 25 samples collected through a mixture of passive and active surveillance.

**Table 3:** Summary showing number of samples, yearly distribution and variant type of triplet expansion and retraction in ORF M159L.

Number of samples	Year distribution	Genome position^a^	Variant type	ORF change and position^b^
10	2018–2025	13,521–13,523	Δ3 NT	Δ1 D161
5	2019–2023	13,518–13,523	Δ6 NT	Δ2 D161‐162
3	2019–2021	13,410–13,415	Δ6 NT	Δ2 D201‐202
1	2022	13,410–13,418	Δ9 NT	Δ DED199‐201
1	2022	13,409–13,423	Δ15 NT	Δ DEDDN199‐203
1	2024	13,426–13,443	Δ18 NT	Δ DDEGED191‐196
1	2018	13,426–13,446	Δ21 NT	Δ EGEDDDD198‐204
2	2021	13,426–13,449	Δ24 NT	Δ DGDDEGED191‐198
8	2023–2025	13,467–13,487	Δ21 NT	Δ HNDEDEN177‐183
6	2020–2021	13,466–13,486	Δ21 NT	Δ EDENNND180‐186
2	2019–2021	13,524	INS6	INS2 D164
7	2018–2021	13,524	INS9	INS3 D164
22	2018–2019	13,524	INS12	INS4 D164
4	2018	13,524	INS15	INS5 D164

^a^Genome position refers to position in MK340973.

^b^Refers to amino acid position within the ORF of M159L.

We observed a notable increase in the detection of samples with triplet deletions and a decrease in the number of triplet insertions in this region post‐2021, 6 years after the initial outbreak. Of the 69 samples with available surveillance data, 15 were obtained through active surveillance and 54 through passive surveillance (Table 4). The percentage of deletions increased after 2021 in both active and passive surveillance while the number of samples demonstrating insertions dropped to 0 % from 2022 on. We also measured the occurrence of multiple SSR variants within individual samples. Multiple events were detected more frequently in actively collected samples (53.3%) than in passive surveillance samples (7.4%). SSR variations in the M159 region were assigned a number code (Mutation ID for haplotyping; Supporting Information 2: Table S2), resulting in 14 designated mutation types. Using this numbering system, 17 haplotypes were identified, comprising both single mutation types and combinations of mutation types. A heatmap comparing province and detected haplotypes was constructed using only isolates with detected mutations (Supporting Information 5: Figure S1). Overall, isolates sharing haplotypes tended to cluster within single or neighbouring provinces, however, haplotype 4 was also detected in geographically distant provinces (Baleares, Caceres, Sevilla and Valladolid) (Supporting Information 5: Figure S1).

**Table 4:** Summary showing number of samples with stable sequences, deletions or insertions, type of surveillance and time period (complete 2018–2025, pre‐ and post‐2021) referring to triplet expansion and retraction in ORF M159L.

Period	Surveillance	*n*	Deletions	Insertions	Stable
2018–2025	Passive	54	14.8%	42.6%	42.6%
2018–2025	Active	15	80%	0%	20%
Pre‐2021	Passive	48	9.2%	45%	43%
Pre‐2021	Active	3	66.7%	0%	33.3%
Post‐2021	Passive	6	100%	0%	0%
Post‐2021	Active	15	100%	0%	0%

The nature of the insertions/deletions resembled microsatellite DNA, or short sequence repeats (SSR). We therefore analysed ha‐MYXV and the MYXV reference genomes for the presence of SSRs.

### 3.5. Microsatellite Identification

**Figure 3:**
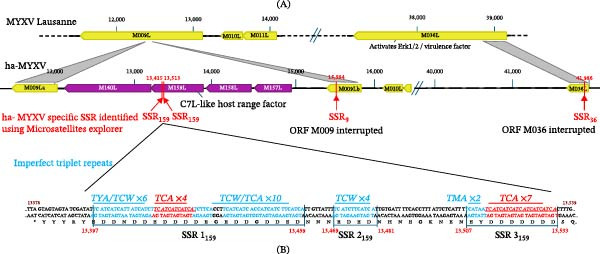
Schematic representation showing relative genome fragments of MYXV Lausanne and ha‐MYXV and the position of four SSR regions in genes potentially implicated in the emergence of ha‐MYXV. (A) Schematic representation of viral *M009L* and *M036L* genome fragments from MYXV Lausanne and ha‐MYXV. Homologous ORFs and ORF fragments are shown in yellow, and the ha‐MYXV‐specific ORFs within Ins‐H1 (*M157L*, *M158L*, *M159L* and *M160L*) are shown in purple. Red lines and arrows indicate the position of the SSRs unique to ha‐MXYV when compared to MYXV Lausanne as identified using microsatellitesexplorer.com. (B) Sequence of the three SSR_159_ regions present in ha‐MYXV *M159L*. Red text indicates the number of TCA repeats, and in blue text the imperfect repeats are indicated. Genome coordinates refer to positions in the ha‐MYXV genome.

Analysis of the ha‐MYXV genome using the Microsatellites Explorer (Microsatellitesexplorer.com; search term ’Myxoma virus’, accessed on 23^rd^ March 2025) revealed it contained five more SSR regions than reference MYXV strain Lausanne (23 vs. 18 SSR regions; Table 5). The 18 SSRs in Lausanne are shared by the sequence of ha‐MYXV with the exception a quadruplet repeated three times within the ORF of *M136R* which is not represented in the ha‐MYXV sequence. However, ha‐MYXV encodes an ambiguous M nucleotide (A or C) in the final position of this SSR (positions 132,801–132,812 in Lausanne) explaining its absence in ha‐MYXV. Interestingly, of the ha‐MYXV and MYXV common SSRs, that present in M064R (M159L homologue), shows a single triplet deletion in ha‐MYXV (Table 5). Of the 5 additional SSR regions in the ha‐MYXV genome, one is present in the ITR before *M000.5L/R* (207–224 present in both ITRs), two are predicted to be present within the *M159L* ORF (nucleotide positions 13,415–13,426 and 13,513–13,533) (Table 5, Figure 3A), one is in the *M009Lb* ORF region (15,584–15,593) (Figure 3A) and one is in the *M036L* region (41,986–41,996) (Figure 3A). As *M159L* is essential for ha‐MYXV replication in hare cell cultures [24] and both *M009L* and *M036L* ORF products are truncated in ha‐MYXV, we focused our analysis on the SSR regions present in these genes.

**Table 5:** Summary of SSR search results from Microsatellites Explorer analysis of MYXV (GenBank accession NC_001132) and ha‐MYXV (GenBank accession MK340973) genomes.

Strain	Gene/ Region	Start	End	Total length	Unit length	Times repeated	Unit sequence
MK340973	**IG0.005L/R**	**207**	**224**	**18**	**6**	**3**	**agaagt**
MK340973	***M159L***	**13,415**	**13,426**	**12**	**3**	**4**	**tca**
MK340973	***M159L***	**13,513**	**13,533**	**21**	**3**	**7**	**atc**
MK340973	***M009Lb***	**15,584**	**15,593**	**10**	**2**	**5**	**ta**
NC_001132	IG14‐15	16,607	16,616	10	1	10	a
MK340973	IG14‐15	19,428	19,437	10	1	10	a
NC_001132	IG17‐18	18,205	18,216	12	4	3	ggta
MK340973	IG17‐18	21,026	21,037	12	4	3	ggta
NC_001132	IG17‐18	18,210	18,241	32	8	4	gtaggtat
MK340973	IG17‐18	21,031	21,062	32	8	4	gtaggtat
NC_001132	*M034L*	35,893	35,904	12	3	4	gtt
MK340973	*M034L*	38,717	38,728	12	3	4	gtt
MK340973	***M036L***	**41,986**	**41,996**	**11**	**1**	**11**	**t**
NC_001132	*M040L*	40,591	40,605	15	3	5	tct
MK340973	*M040L*	43,419	43,433	15	3	5	tct
NC_001132	***M064R***	**60,117**	**60,131**	**15**	**3**	**5**	**gaa**
MK340973	***M064R***	**62,949**	**62,960**	**12**	**3**	**4**	**gaa**
NC_001132	*M068R*	62,320	62,331	12	3	4	acg
MK340973	*M068R*	65,149	65,160	12	3	4	acg
NC_001132	*M080R*	77,516	77,527	12	4	3	gatc
MK340973	*M080R*	80,345	80,356	12	4	3	gatc
NC_001132	*M080R*	77,643	77,654	12	4	3	tacg
MK340973	*M080R*	80,472	80,483	12	4	3	tacg
NC_001132	*M085R*	83,762	83,771	10	2	5	at
MK340973	*M085R*	86,591	86,600	10	2	5	at
NC_001132	*M086L*	85,820	85,831	12	3	4	aac
MK340973	*M086L*	88,649	88,660	12	3	4	aac
NC_001132	*M107L*	103,905	103,914	10	2	5	ac
MK340973	*M107L*	106,734	106,743	10	2	5	ac
NC_001132	*M108R*	104,215	104,226	12	3	4	ttc
MK340973	*M108R*	107,044	107,055	12	3	4	ttc
NC_001132	*M111R*	107,095	107,106	12	3	4	gaa
MK340973	*M111R*	109,924	109,935	12	3	4	gaa
NC_001132	*M116L*	113,678	113,689	12	3	4	acg
MK340973	*M116L*	116,507	116,518	12	3	4	acg
NC_001132	*M118L*	114,636	114,647	12	4	3	gttc
MK340973	*M118L*	117,465	117,476	12	4	3	gttc
NC_001132	*M136R*	132,801	132,812	12	4	3	aaat
MK340973	*M136R*	140,002	140,013	12	4	3	aaat
NC_001132	*M141R*	137,192	137,203	12	3	4	agt
MK340973	*M141R*	140,002	140,013	12	3	4	agt
MK340973	**IG0.005L/R**	**164,335**	**164,352**	**18**	**6**	**3**	**ctactt**

*Note:* Ha‐MYXV unique SSR shown in bold type and orange fill. The SSR region in M064R is shown in bold type and grey fill.

Abbreviation: IG, intergenic region.

### 3.6. SSR Within the M159 ORF

Microsatellites Explorer predicts two SSRs in the *M159L* ORF separated by 87 nts. The first has a total length of 12 nts and consists of four trinucleotide (TCA) repeats (bp 13,415–13,426) (Table 5; Figure 3B shown in red font). The second SSR is 21 nts in length and contains seven copies of the ATC TCA (bp 13,513–13,533) (Table 5; Figure 3B). As *M159L* is in the L orientation, the ATC triplet becomes the GAT codon when read in frame. Both SSRs encode polyD (aspartic acid) regions at the C‐terminal region of M159. While Microsatellites Explorer identified perfect triplet repeats, a closer analysis of the sequence and allowing for imperfect triplets (T‐A or T‐C within the triplets) (blue font; Figure 3B) lets us define three SSR regions in *M159L*, which we have denominated SSR1_159_, SSR2_159_ and SSR3_159_ (Figure 3B). SSR1_159_ and SSR2_159_ are separated by a nine nts spacer sequence (TTGTTATTT), and SSR2_159_ and SSR3_159_ are separated by a spacer region of 27 nts. The first nine nts of spacer 2 (TTGTGATTT) resemble the spacer 1 region with a single nt change. The following 18 nts of the spacer 2 region contain a stretch of nts similar to the SSR_159_ regions with a number of mutations (TCACCTTTATTCTCATTT).

### 3.7. Enrichment and Reduction of SSRs in *M159L*

**Figure 4:**
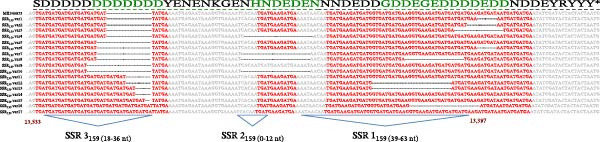
Multiple sequence alignment of representative samples of each SSR variant type (17 in total) detected in this study. The predicted ORF sequence is shown above the alignment with amino acids affected by deletions or insertions shown in green. SSR1, 2 and 3 are shown in red text, and total lengths are indicated below the alignment. Deleted regions are indicated with a dash (‐). Conserved flanking sequences are in grey font.

**Figure 5:**
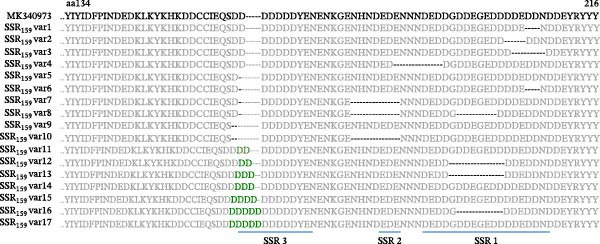
Multiple sequence alignment of predicted amino acid sequences from representative samples of each SSR variant type detected in this study. The predicted ORF sequence of reference ha‐MYXV strain (GenBank accession MK340973) is shown above the alignment in black font. SSR regions 1, 2 and 3 are indicated by the blue lines. Conserved sequences are shown in grey text. Predicted amino acid changes are shown in green font. Deleted amino acids are indicated with a bold dash (**-**).

Analysis of the multiple sequence alignments of the 106 ha‐MYXV field samples in the SSR_159_ region revealed 17 variant types, each comprised of combinations of mutations in the three SSRs (Figure 4; Supporting Information 2: Table S2). Nine samples showed variations in SSR1_159_ with deletions ranging from 6 to 24 nts (total of eight variant types) (Supporting Information 2: Table S2; Figure 4). Fourteen samples (four variant types) had deletions of 21 nts in the SSR2_159_. The deletion of SSR2_159_ was complete in these isolates and included partial deletion of spacer 1 and 2 sequences (Supporting Information 2: Table S2; Figure 4). SSR3_159_ showed the most variation, with deletions of three or six nts (15 samples; six variant types) and insertions ranging in length from 6 to 15 nts in 35 samples (seven variant types). Of the samples analysed, 10 had combinations of two mutation types, and one sample (Vall/24‐25IT_8) combined three SSR_159_ mutation types (Supporting Information 2: Table S2). The predicted effect on the amino acid sequences of the 17 variant types compared to ha‐MYXV is depicted in Figure 5. Extensions and contractions of poly D (aspartic acid) tracts can be observed in the predicted ORFs in the region corresponding to the C‐terminal region of the protein (Figure 5). Samples collected from infected rabbits included deletions in SSR1, SSR2 and SSR3 (Δ6, Δ21 and Δ6 nts, respectively, corresponding to variant types 1 and 10).

**Figure 6:**
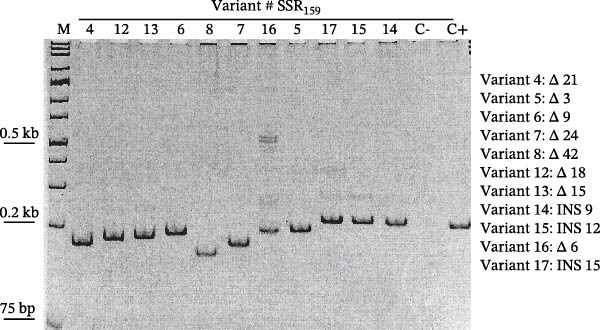
Polyacrylamide gel electrophoresis analyses of PCR amplicons obtained following amplification of the SSR_159_ region from 11 representative variants. Ha‐MYXV isolate Tol08‐18 substrain L3_031 DNA was used as a positive control (C+). Variant identification numbers and SSR region data compared to positive controls are indicated in the box. Variant numbers (shown in the key and above each lane) correspond to the sequence variants defined in Figure 4. The size of each insertion or deletion is specified in nucleotides (e.g. INS 9 = insertion of 9 nt, Δ 3 = deletion of 3 nt).

Given these observations, we designed a conventional PCR to target the SSR_159_ region and allow differentiation of mutation types present in representative isolates by gel electrophoresis. A selection of isolates containing 11 identified variants was analysed by polyacrylamide gel electrophoresis following the PCR (Figure 6). Variants with insertions or deletions with respect to the ha‐MYXV L3 substrain were distinguishable using this PCR: for example insertions (INS) or deletions (Δ) of three nts or more are distinguishable.

### 3.8. Variation in SSR Within Region M009Lb

**Figure 7:**
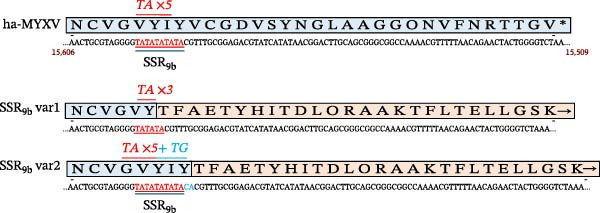
Schematic representation of SSR9b variant sequence. Ha‐MYXV SSR_9b_ is depicted (15,606 to 15,509) in reverse orientation to show the amino acid sequence. The premature stop codon is marked with an asterisk. Indicated in red text and bold type are the number of copies of the TA sequence in each variant. Above the nucleotide sequence of each SSR_9b_ variant, the predicted ORFs are indicated with the original sequence shown in blue and the mutated ORF shown in orange.

ORF *M009L* (nt 11,601–13,130 MYXV Lausanne) is divided at position 12236 into two parts, *M009La* and *M009Lb*, respectively, due to the insertion of Ins‐H1 [23]. Microsatellites Explorer identified an SSR of five copies of the dinucleotide repeat TA within ORF *M009Lb* (Table 5; Figure 7) not present in MYXV Lausanne, which we have denominated SSR_9 b_. This is the site of the TATA insertion detected in ha‐MYXV that is predicted to disrupt ORF *M009L* at amino acid 121 (leading to truncation and loss of aa 121–510; [23] independently of the Ins‐H1 region. We analysed a subset of the 106 samples for sequence variations in the SSR_9b_ region.

Sequence analysis of 69 samples of the SSR_9b_ region revealed three variant types circulating in the field. Twenty‐five (25) samples were the same as the reference ha‐MYXV strain, 39 samples had a reduction in the SSR from 5xTA to 3xTA, and 5 samples had 5xTA plus an insertion of TG (Figure 7). The four base pair deletion would lead to the extension of the ORF9b by an additional 153 amino acids (Figure 7), terminating 48 nts prior to the *M160L* ORF, while the additional TG mutation would lead to the same change in ORF extension with the inclusion of two additional amino acids in the SSR region (Figure 7).

### 3.9. Variation in SSR Within Region M036L

**Figure 8:**
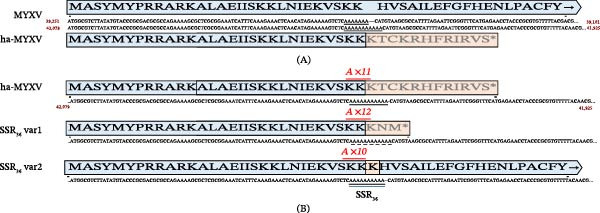
Schematic representation of SSR_36_ variant sequences. (A) The sequences encompassing the ha‐MYXV SSR_36_ are compared to the same region in MYXV (41,925–42,079 and 39,101–39,251, respectively) in reverse orientation to show the predicted amino acid sequence of each ORF. The premature stop codon is marked with an asterisk. (B) Ha‐MYXV SSR_36_ and two variant sequences (var1 and var2) are depicted with the length of each SSR indicated in red text and bold type (i.e. the number of copies of the A sequence repeat in each variant). Above the nucleotide sequence of each SSR_36_ variant, the predicted ORFs are indicated with the original sequence shown in blue and the mutated ORF shown in orange.

Ha‐MYXV ORF *M036L* (ha‐MXYV 40,033–41,781) is truncated when compared to MYXV‐Lau *M036L* due to a four‐nucleotide insertion (TTTT) at position 41,986 (39,164 in MYXV‐Lau) [23]. Microsatellites Explorer identified an SSR of 11 copies of the nucleotide T repeated within the *M036L* gene region of ha‐MYXV (Table 5; Figure 8A).

Sequencing of 68 samples in the *M036L* SSR region found three variant types. Twelve field isolates showed a single T deletion with respect to ha‐MYXV in SSR_36_, reducing the SSR from 11xT to 10xT. The predicted effect on the ORF is shown in Figure 8. Twenty‐eight (28) samples had the same sequence as ha‐MYXV, while 28 isolates showed a single T insertion in the SSR_36_ (extending it to 12xT). While the 11xT and 12xT SSRs lead to early truncation of the M036L, the 10xT is predicted to re‐establish the ORF to the MYXV‐Lau *M036L* ORF with the addition of a single K residue at the SSR site.

## 4. Discussion

Gene loss and fragmentation have been shown to be major factors in chordopoxvirus evolution [32–35]. The introduction of premature stop codons (early stop mutations [ESMs]) is often a consequence of the variability in the number and nature of SSRs and may facilitate adaptation to novel hosts following a species jump [36].

In this study, we undertook an analysis of the stability of four novel genes from ha‐MYXV responsible for a myxomatosis epizootic in Iberian hare populations following a species jump. Of the four ha‐MYXV‐specific genes, neither *M157L* nor *M158L* suffered mutations in any of the 106 samples analysed. The *M157L* homologue in vaccinia virus (VACV) encodes the J1R (*M060R* in MYXV) protein, a late viral protein that is required for immature virion formation [37]. The *M158L* gene encodes a viral thymidine kinase protein homologous to J2R in VACV, the deletion of which causes attenuation [38]. In general, the *M160L* gene was highly conserved throughout the samples. This gene encodes a regulatory poly(A) polymerase subunit J3R homologue. J3R interacts with viral protein H4L through its C‐terminal residues to form a poly(A) polymerase [39]. In our study, eight isolates were found to have a SNP that would render this activity lost, as the truncation of the ORF would lead to the loss of the C‐terminal region (with Δ343, Δ320 and Δ215 amino acids of the 351 amino acid total protein length). These mutations would presumably be compensated for by the presence of *M065R* in the central region of the ha‐MYXV genome, allowing the virus to maintain its replication capacity and virulence in the absence of a functional M160 poly(A) polymerase subunit. In these strains the alternative pathways or redundant genes within the viral genome may mitigate the impact of disruptive mutations, ensuring viral viability. Within the *M160L* gene we also observed that ambiguous nts previously reported in the L3 isolate had been fixed. These ambiguous sites were previously detected using next‐generation sequencing, while the sequences in this study were generated by PCR. This discrepancy should be treated with caution. Although no mixed peaks were observed at these sites in the Sanger sequencing data, this does not rule out sequence heterogeneity, including low copy number variants, that may be missed.

M159 is a C7L protein homologue and has previously been shown to be required for ha‐MYXV replication in hare cells in vitro [24]. M159 showed the most variation in the isolates analysed in this study. The gene suffered alterations in the form of triplet nucleotide insertions and deletions, always maintaining the predicted ORF. The molecular mechanism of SSR expansion and retraction has not been determined, although the literature suggests that slippage of the DNA polymerase during replication is the cause and that these regions may serve as “hot spots” for recombination [34, 36]. Interestingly, the regions that suffered the mutations correspond to the highly negatively charged C‐terminal tail and are not predicted to interfere with the five VACV C7 conserved residues (K24, D51, W52, E54 and F79) or to form part of the characteristic C7 structure indicating that the predicted function is likely conserved [24]. The C‐terminal tail region is not present in the 3D structural model [40] and is highly variable in the poxvirus C7L family but does share the characteristic of containing many acidic amino acids [40, 41]. Given this information, we cannot speculate on any specific effect that the SSR expansion and retraction may have on protein function. However, the presence of these regions in the variable tail region are tolerated during virus replication and infection. It is interesting to speculate that the extensions and reductions in the length of this cytoplasmic tail may play a role in the adaptation of this virus to its novel host and may even facilitate further attempts to jump to closely related species (e.g. the European brown hare). Indeed, recent reports have confirmed the presence of ha‐MYXV in this species in Spain [26] and north western Europe [27]. Our data show that there was a rapid expansion of the SSR_159_ region in a large number of isolates from Iberian hares in the years following ha‐MYXV emergence. These samples were collected through passive surveillance upon finding dead and diseased animals. Samples collected in the latter years of this study (2023–2025) generally contain SSR_159_ regions with deletions. These samples were collected through both active and passive surveillance and include samples from animals with no myxomatosis symptoms. A study of 28 naturally infected Iberian hares demonstrated pathological lesions following infection by ha‐MYXV [42]. Although there is no further data regarding the pathogenesis or virulence of ha‐MYXV in the Iberian hare, it is tempting to speculate that the appearance of animals with no symptoms may imply that attenuated strains of the virus are evolving, as has been described for MXYV following release in Australia and Europe (reviewed by Kerr [43]). Future studies may provide insights into the events of expansion and contraction and possible links with virulence changes. Members of the C7L protein family are host range factors that play an important role in host specificity and the virus’s ability to evade or counteract the defences of the infected host immune system (reviewed in Liu et al. [44]). Variations in host range genes have been linked to poxvirus outbreaks. For example, during a zoonotic outbreak of buffalopox virus, four mutations in the C7L gene and one in the B5R gene were related to buffalo infections [45, 46] that were not observed in human or cow isolates. However, the complex nature of the poxvirus genome and the vast array of host range factors make it difficult to define links between single mutations and functional data.

The identification of SSR regions within the M159 ORF led us to investigate the presence of further SSRs in the genome of ha‐MYXV. The prediction of two additional SSRs occurring in key genome locations and leading to ESMs (SSR_9_ and SSR_36_) is compelling.

In MYXV‐Lau *M009L* encodes a putative ubiquitin (Ub) E3 ligase of 599 aa with an N‐terminal BTB‐BACK domain followed by four Kelch motifs [47]. This protein is homologous to MYXV proteins M006 and M008, both present in the MYXV genome in two copies in the ITR region (L and R). ORF *M009L* has undergone multiple disruptions during MYXV evolution as observed in Australian strains [15, 48, 49] and was also lost in the Californian MSW strain [50]. Only a small group of Australian viruses (six viruses belonging to lineage A) retained this ORF intact [51]. In Europe, five strains have been detected as having deletions in ORF M009; FLI‐H (GenBank Accession KP723390), 2604 (GenBank Accession KP723389) and 3207 (GenBank Accession KP723388) have deletions of 162 amino acids; MAV (GenBank Accession KP723391) has lost 408 amino acids and there is a complete deletion in Munich‐1 (GenBank Accession KP723387) [49]. The attenuated Spanish MYXV strain 6918 (GenBank accession EU552530) shows a deletion of 10 bp, resulting in a frameshift that divides the gene into two putative ORFs [52]. In ha‐MYXV the Ins‐H1 region interrupts *M009L*, also dividing it into two parts (designated *M009La* and *M009Lb*) [23]. These findings suggest that this gene is not a critical virulence factor for MYXV, and this may have been determinant for the insertion and maintenance of Ins‐H1 within this gene.

In MYXV Lausanne, the *M036L* gene is 2043 bp in length (37,209–39,251 bp); however, in ha‐MYXV, the insertion of four thymidines at position 41,986 bp results in disruption of this ORF and the generation of SSR_36_ (change from 7 to 11xT) with the ORF region. Mutations in the *M036L* gene have been observed in the Australian isolate (BRK) (GenBank Accession JX565562) [48], the Spanish attenuated strain 6918 [52], and isolates from the UK (Sussex [GenBank Accession KC66084] and Nottingham [GenBank Accession JX565572], both attenuated, and Perthshire lineages 1 and 2 [GenBank Accession KY548792‐KY548801]) [53]. MYXV Lausanne does not include an SSR in this region, as the minimum 8 nt repeat is not reached as defined by Chambers and MacAvoy [1]. Of the field isolates analysed, 40 showed variations in this SSR. Twelve isolates have the SSR reduced by a single thymidine, whereas 28 strains contain an additional thymidine inserted in this region. MYXV *M036L* is a homologue of the VACV *O1L* [14], the protein product of which enhances signalling through Erk1/2 in human cells in vitro [54]. Intriguingly, disruption of the Erk signalling leads to permissiveness of primary mouse embryo fibroblasts to MYXV [55]. The role of *M036L* in hare infection with ha‐MYXV remains to be determined.

It has previously been observed that 65% of ESM are associated with SSRs in poxviruses [36]. The truncation of ORFs *M009L* and *M036L* in ha‐MYXV [22, 23] is associated with the appearance of SSRs. Both *M009L* and *M036L* have previously been observed to be interrupted in a variety of Spanish MYXV isolates [29, 52]. Such mutations that led to ESMs may have paved the way for the appearance of ha‐MYXV, facilitating the species jump.

The limited number of European rabbit samples analysed in this study does not allow us to draw conclusions on the selection of ha‐MYXV‐specific sequences or transmission of ha‐MYXV in this species, and further studies will be needed to address these issues. However, the identification of SSR variation in M159 allowed the designation of 17 different variants or haplotypes. Monitoring such variation may provide a basis for future research on viral transmission paths and regional adaptive evolution in ha‐MYXV. Our data indicate that haplotypes generally clustered in neighbouring provinces; however, identical haplotypes were also observed in geographically distant provinces. This may indicate long‐distance dissemination or, alternatively, independent emergence of the same variant types. In this context, the potential emergence of virus variants and changes in virulence in lagomorph species should be monitored.

The origin of the region Ins‐H1 still remains to be identified. Previous phylogenetic analyses revealed similarities between the first characterised ha‐MYXV sequences and published poxvirus sequences [22, 23]. The low mutation rate observed for the ORFs studied in the present study is somewhat surprising since the sequence already seems to be well adapted to its novel host, thus indicating a leporid virus origin of the insert Ins‐H1 sequence, leading us to speculate that there is an as yet unidentified hare poxvirus circulating in the Iberian hare population. Alternatively, ha‐MYXV gained this region from a non‐leporid virus origin and has been circulating for a long enough period of time to adapt to its current sequence. The high conservation of *M157L*, *M158L* and *M160L* contrasts with the variability described in this study for the SSR regions. Our findings highlight the need for continued epidemiological and molecular surveillance in order to better understand virus evolution following this cross‐species virus transmission event.

The MYXV and ha‐MYXV genomes are large and highly conserved, facilitating routine diagnostic confirmation using a single gene target. However, the documented shift in species tropism is epidemiologically relevant and requires continued monitoring. Our findings suggest that monitoring of the polymorphic SSR regions, particularly SSR159 and SSR36, may provide markers to determine variation in field samples. Monitoring the emergence of new variants will help support conservation efforts for the populations of Iberian and European brown hares and prevention and control of the disease on rabbit farms.

In conclusion, the information provided by this study defines genome regions that may be targeted in future molecular epidemiological studies regarding ha‐MYXV. Our data also demonstrate hypervariable microsatellite regions that may play a role in the evolution of ha‐MYXV and point to the need for continued active surveillance of this pathogen.

## Author Contributions

Conceptualisation: K. P. Dalton, I. García‐Bocanegra, F. Parra and J. M. Martín‐Alonso. Sample collection, methodology and analysis: A. Menéndez‐Manjón, B. Cardoso, S. Castro‐Scholten, M. Agüero, M. Duran, D. Buitrago, J. Abrantes, D. Cano‐Terriza, D. Jiménez‐Martín, L. Camacho‐Sillero, I. Nicieza, S. Arenas‐Vicente, I. Calonge‐Sanz, F. Parra, A. L. Alvarez and P. Domínguez. Project administration and investigation: K. P. Dalton, I. García‐Bocanegra and J. M. Martín‐Alonso. Writing – original draft: K. P. Dalton, A. Menéndez Manjón and I. García‐Bocanegra. Writing – review and editing: all authors.

## Funding

K. P. Dalton and J. M. Martín‐Alonso were supported by Grant MCI‐21‐PID2020‐120349RB‐100 and A. Menéndez Manjón by Grant PRE2021‐100644 for predoctoral contracts for doctoral training cofinanced by FEDER. K. P. Dalton is supported by the Spanish Ministry of Science and Innovation (Grant PID2024‐161840OB‐100). This work was also supported by the financial aid of research grants funded by the Spanish Ministry of Science and Innovation (projects: Iber‐LagoHealth; REF: PID2023‐151954NB‐I00; LagoHealth and REF: PID2019‐111080RB‐C21) and by the CIBER ‐ Consorcio Centro de Investigación Biomédica en Red (Grant CB2021/13/00083), the Instituto de Salud Carlos III and the Ministerio de Ciencia e Innovación and Unión Europea–NextGenerationEU. D. Jiménez Martín was supported by a FPU grant (Grant FPU22/03649) funded by the Spanish Ministry of Universities. We acknowledge Projects SV‐PA‐21‐AYUD/2021/51288 from the Principado de Asturias (Spain); SBPLY/17/180501/00051 from the Junta de Comunidades de Castilla‐La Mancha and the European Regional Development Fund and I‐FEDEXCAZA202013 from the Federación Extremeña de Caza. We are thankful to INTERCUN for the financial and logistical support. B. Cardoso was supported by the FCT (Grant 2020.04872.BD).

## Consent

The authors have nothing to report.

## Conflicts of Interest

The authors declare no conflicts of interest.

## Supporting Information

Additional supporting information can be found online in the Supporting Information section.

## Supporting information


**Supporting Information 1** Table S1: Summary table of sample data indicating isolate name, year of collection, province, species and type of surveillance (active or passive).

**Supporting Information 2** Table S2: Table showing details of the mutations detected in each isolate, including Gene affected, genome position, variant sequences and haplotype identification number.

**Supporting Information 3** Table S3: A heatmap matrix indicating percentage identity between all 106 samples.

**Supporting Information 4** Table S4: Table showing ha‐MYXV isolate name, province, year of collection and the percent identity when compared with reference isolate MK340973.1.

**Supporting Information 5** Figure S1: Heatmap showing the distribution of detected haplotypes across provinces based on SSR 159 sequence analysis. Each mutation type was designated a number, and combinations of mutation types present in samples were used to define individual haplotypes. Haplotype numerical codes are defined in Supporting Table 2 and are shown on the *X*‐axis, province names are shown on the *Y*‐axis.

## Data Availability

All data and material are available upon reasonable request.
