# Supplementary material for: Enrichment and Reduction of Microsatellite Regions in the Myxoma Virus Genome Following Species Jump to the Iberian Hare (Lepus granatensis)
Source: Transbound Emerg Dis. 2026 Apr 18;2026:3847131. doi: 10.1155/tbed/3847131 (PMC13091234; doi:10.1155/tbed/3847131)
Supplement: Supplementary file 1 — Supporting Information 1 Table S1: Summary table of sample data indicating isolate name, year of collection, province, species and type of surveillance (active or passive). [file TBED-2026-3847131-s001.docx]

Supplementary Table 1. Summary of sample data including isolate name, year, province, species and surveillance type.

| **ha-MYXV isolate** | **Year** | **Province** | **Species** | **Surveillance type** |
| --- | --- | --- | --- | --- |
| Alm/18-L57 | 2018 | Almeria | Iberian hare | Passive |
| Alm18-L56 | 2018 | Almeria | Iberian hare | Passive |
| Bad/18-2249_1 | 2018 | Badajoz | Iberian hare |  |
| Bad/18-2281_3 | 2018 | Badajoz | Iberian hare |  |
| Bal/18-3532_5 | 2018 | Baleares | Iberian hare |  |
| Cad/18-L22 | 2018 | Cadiz | Iberian hare | Passive |
| Cad/18-L24 | 2018 | Cadiz | Iberian hare | Passive |
| Cad/18-L25 | 2018 | Cadiz | Iberian hare | Passive |
| CiudR/18-L26 | 2018 | Ciudad Real | Iberian hare | Passive |
| CiudR/18-L27 | 2018 | Ciudad Real | Iberian hare | Passive |
| Cord/18-L10 | 2018 | Cordoba | Iberian hare | Passive |
| Cord/18-L11 | 2018 | Cordoba | Iberian hare | Passive |
| Cord/18-L12 | 2018 | Cordoba | Iberian hare | Passive |
| Cord/18-L15 | 2018 | Cordoba | Iberian hare | Passive |
| Cord/18-L16 | 2018 | Cordoba | Iberian hare | Passive |
| Cord/18-L17 | 2018 | Cordoba | Iberian hare | Passive |
| Cord/18-L18 | 2018 | Cordoba | Iberian hare | Passive |
| Cord/18-L19 | 2018 | Cordoba | Iberian hare | Passive |
| Cord/18-L20 | 2018 | Cordoba | Iberian hare | Passive |
| Cord/18-L21 | 2018 | Cordoba | Iberian hare | Passive |
| Cord/18-L9 | 2018 | Cordoba | Iberian hare | Passive |
| Cuen/18-ha36 | 2018 | Cuenca | Iberian hare | Passive |
| Gran/18-L67 | 2018 | Granada | Iberian hare | Passive |
| Gran/18-L68 | 2018 | Granada | Iberian hare | Passive |
| Gran/18-L69 | 2018 | Granada | Iberian hare | Passive |
| Huel/18-L70 | 2018 | Huelva | Iberian hare | Passive |
| Huel/18-L71 | 2018 | Huelva | Iberian hare | Passive |
| Huel/18-L72 | 2018 | Huelva | Iberian hare | Passive |
| Jaen/18-1844_4 | 2018 | Jaen | Iberian hare |  |
| Jaen/18-L30 | 2018 | Jaen | Iberian hare | Passive |
| Jaen/18-L31 | 2018 | Jaen | Iberian hare | Passive |
| Jaen/18-L33 | 2018 | Jaen | Iberian hare | Passive |
| Jaen/18-L34 | 2018 | Jaen | Iberian hare | Passive |
| Jaen/18-L35 | 2018 | Jaen | Iberian hare | Passive |
| Jaen/18-L36 | 2018 | Jaen | Iberian hare | Passive |
| Jaen/18-L37 | 2018 | Jaen | Iberian hare | Passive |
| Jaen/18-L38 | 2018 | Jaen | Iberian hare | Passive |
| Mad/18-2255_3 | 2018 | Madrid | Iberian hare |  |
| Mal/18-L39 | 2018 | Malaga | Iberian hare | Passive |
| Mal/18-L40 | 2018 | Malaga | Iberian hare | Passive |
| Mal/18-L41 | 2018 | Malaga | Iberian hare | Passive |
| Mal/18-L42 | 2018 | Malaga | Iberian hare | Passive |
| Mal/18-L44 | 2018 | Malaga | Iberian hare | Passive |
| Mal/18-L45 | 2018 | Malaga | Iberian hare | Passive |
| Mal/18-L47 | 2018 | Malaga | Iberian hare | Passive |
| Mal/18-L48 | 2018 | Malaga | Iberian hare | Passive |
| MK340973  Ha-MYXV Tol/18 L3 | 2018 | Toledo | Iberian hare |  |
| Mur/18-2388_1 | 2018 | Murcia | Iberian hare |  |
| Sev/18-L54 | 2018 | Sevilla | Iberian hare | Passive |
| Sev/18-L55 | 2018 | Sevilla | Iberian hare | Passive |
| Sev/18-L89 | 2018 | Sevilla | Iberian hare | Passive |
| Sor/18-3373_3 | 2018 | Soria | Iberian hare |  |
| Tol/18-1981_4 | 2018 | Toledo | Iberian hare |  |
| Tol/18-2080_1 | 2018 | Toledo | Rabbit |  |
| Ala/19-2678_1 | 2019 | Álava | Iberian hare |  |
| Alb/19-1326_4 | 2019 | Albacete | Iberian hare |  |
| Avil/19-2741_10 | 2019 | Ávila | Iberian hare |  |
| Avil/19-2815_1 | 2019 | Ávila | Iberian hare |  |
| Bad/19-2485_4 | 2019 | Badajoz | Iberian hare |  |
| Bal/19-2789_2 | 2019 | Baleares | Iberian hare |  |
| Cac/19-2681_8 | 2019 | Cáceres | Iberian hare |  |
| Cad/19-2534_1 | 2019 | Cádiz | Iberian hare |  |
| Cord/19-2534_8 | 2019 | Cordoba | Iberian hare |  |
| Cuen/19-2467_22 | 2019 | Cuenca | Iberian hare |  |
| Cuen/19-3776 | 2019 | Cuenca | Rabbit |  |
| Gran/19-2667_5 | 2019 | Granada | Iberian hare |  |
| Jaen/19-ha30 | 2019 | Jaen | Iberian hare | Passive |
| Mal/19-2534_3 | 2019 | Malaga | Iberian hare |  |
| Mur/19-3334 | 2019 | Murcia | Rabbit DOMESTICO |  |
| Pal/19-2865_4 | 2019 | Palencia | Iberian hare |  |
| Ter/19-2879_4 | 2019 | Teruel | Iberian hare |  |
| Val/19-1190_1 | 2019 | Valencia | Iberian hare |  |
| Vall/19-2585_2 | 2019 | Valladolid | Iberian hare |  |
| Vall/19-2614_1 | 2019 | Valladolid | Iberian hare |  |
| Vall/19-2614_2 | 2019 | Valladolid | Iberian hare |  |
| Vall/19-2614_3 | 2019 | Valladolid | Iberian hare |  |
| Zara/19-2690_1 | 2019 | Zaragoza | Iberian hare |  |
| Avil/20-ha7 | 2020 | Ávila | Iberian hare |  |
| Cord/20-ha33 | 2020 | Cordoba | Iberian hare | Passive |
| Leon/20-ha6 | 2020 | Leon | Iberian hare |  |
| Mur/20-ha24 | 2020 | Murcia | Iberian hare | Passive |
| Sev/20-ha31 | 2020 | Sevilla | Iberian hare | Passive |
| Zamo/20-ha8 | 2020 | Zamora | Iberian hare |  |
| Bad/21-ha17 | 2021 | Badajoz | Iberian hare | Active |
| Bad/21-ha18 | 2021 | Badajoz | Iberian hare | Active |
| Bad/21-ha20 | 2021 | Badajoz | Iberian hare | Active |
| Bad/21-ha25 | 2021 | Badajoz | Iberian hare | Active |
| Bad/21-ha27 | 2021 | Badajoz | Iberian hare | Active |
| Bad/21-ha28 | 2021 | Badajoz | Iberian hare | Active |
| Bad/21-ha34 | 2021 | Badajoz | Iberian hare | Active |
| Cac/21-ha21 | 2021 | Cáceres | Iberian hare | Active |
| CiudR/21-ha9 | 2021 | Ciudad Real | Iberian hare |  |
| Cuen/21-ha22 | 2021 | Cuenca | Iberian hare | Passive |
| Cuen/21-ha29 | 2021 | Cuenca | Iberian hare | Passive |
| Jaen/21-ha19 | 2021 | Jaen | Iberian hare | Active |
| Mal/21-ha23 | 2021 | Malaga | Iberian hare | Passive |
| Sala/21-ha5 | 2021 | Salamanca | Iberian hare |  |
| Bad/22-3746_4 | 2022 | Badajoz | Iberian hare |  |
| Cord/22-ha16 | 2022 | Cordoba | Iberian hare | Passive |
| Alm23-25IT_17 | 2023 | Almeria | Rabbit | Active |
| Cord/23-25IT-14 | 2023 | Cordoba | Rabbit | Active |
| Vall/24_1_25IT_8 | 2024 | Valladolid | Iberian hare | Active |
| Vall/24-2_25IT_9 | 2024 | Valladolid | Iberian hare | Active |
| Tol/25_25IT_7 | 2025 | Toledo | Iberian hare | Passive (granja) |
| Tol/25-25IT_4 | 2025 | Toledo | Iberian hare | Passive (granja) |
| Vall/25-25IT_11 | 2025 | Valladolid | Iberian hare | Active |
| Vall/25-25IT_12 | 2025 | Valladolid | Iberian hare | Active |
