# Supplementary material for: Enrichment and Reduction of Microsatellite Regions in the Myxoma Virus Genome Following Species Jump to the Iberian Hare (Lepus granatensis)
Source: Transbound Emerg Dis. 2026 Apr 18;2026:3847131. doi: 10.1155/tbed/3847131 (PMC13091234; doi:10.1155/tbed/3847131)
Supplement: Supplementary file 2 — Supporting Information 2 Table S2: Table showing details of the mutations detected in each isolate, including Gene affected, genome position, variant sequences and haplotype identification number. [file TBED-2026-3847131-s002.docx]

Supplementary Table 2. Summary of variants detected in the *M159L* and *M160L* ha-MYXV genes inlcuding comparision to reference genome and changes predicted to affect ORFs.

| ha-MYXV isolates | Gene | Genome position^a^ | Ref. seq | Variant seq. | Mutation ID for haplotyping | ORF and position^b^ |
| --- | --- | --- | --- | --- | --- | --- |
| Jaen/18-L33; Vall/19-2614_2; CiudR/21-ha9 ; Cuen/21-ha22; Vall/24-25IT_8; Vall/24-25IT_9; Tol/25-25IT_4; Tol/25-25IT_7; Vall/25-25IT_11; Vall/25-25IT_12; | M159L | 13,521- 13,523 | ATC | Δ3 NT | 1 | Δ1 D161 |
| Avil/19-2741_10; Zamo/20-ha8; Jaen/21-ha19; Cord/23-25IT_14; Alm/23-25IT_17 | M159L | 13,518- 13,523 | ATCATC | Δ6 NT | 2 | Δ2 D161-162 |
| Cuen/19-3776; Cuen/19-2467_22; Cuen/21-ha22 | M159L | 13,410- 13,415 | ATCATC | Δ6 NT | 9 | Δ2 D201-202 |
| Bad/22-3746_4 | M159L | 13,410- 13,418 | ATCATCTTC | Δ9 NT | 10 | Δ DED199-201 |
| Cord/22-ha16 | M159L | 13,409- 13,423 | TATCATCTTCATCAT | Δ15 NT | 11 | Δ DEDDN199-203 |
| Vall/24-25IT_8 | M159L | 13,426- 13,443 | TCATCTTCACCTTCATCA | Δ18 NT | 12 | Δ DDEGED191-196 |
| Jaen/18-L37 | M159L | 13,426- 13,446 | TCATCTTCACCTTCATCATCA | Δ21 NT | 14 | Δ EGEDDDD198-204 |
| Bad/21-ha17; Bad/21-ha20 | M159L | 13,426- 13,449 | TCATCTTCACCTTCATCATCACCA | Δ24 NT | 13 | Δ DGDDEGED191-198 |
| Cord/23-25IT_14; Alm/23-25IT_17 ; Vall/24-25IT_8; Vall/24-25IT_9; Tol/25-25IT_4; Tol/25-25IT_7; Vall/25-25IT_11; Vall/25-25IT_12; | M159L | 13,467- 13,487 | ATTTTCATCTTCATCATTGTG | Δ21 NT | 8 | Δ HNDEDEN177-183 |
| Mur/20-ha24; Bad/21-ha18; Cac/21-ha21; Bad/21-ha25; Cuen/21-ha29; Sala/21-ha5 | M159L | 13,466- 13,486 | TATTTTCATCTTCATCATTGT | Δ21 NT | 7 | Δ EDENNND180-186 |
| Bad/19-2485_4; Bad/21-ha17 | M159L | 13,524 | - | INS6 ATCATC | 3 | INS2 D164 |
| Sev/18-L54; Vall/19-2585_2; Vall/19-2614_1; Vall/19-2614_3; Cac/19-2681_8; Bal/19-2789_2; Bad/21-ha20 | M159L | 13,524 | - | INS9 ATCATCATC | 4 | INS3 D164 |
| Jaen/18-L36; Gran/18-L68; Mal/18-L44; Mal/18-L39; Mal/18-L40; Mal/18-L42; Mal/18-L45; Mal/18-L41; Jaen/18-L30; Gran/18-L69; CiudR/18-L26; Cord/18-L11; Cord/18-L21; Jaen/18-L34; Jaen/18-L38; Mal/18-L47; Mal/18-L48; Cord/18-L9;  Jaen/18-1844_4; Bad/18-2281_3; Cord/19-2534_8; Jaen/18-L31; | M159L | 13,524 | - | INS12 ATCATCATCATC | 5 | INS4 D164 |
| Jaen/18-L37; Alm/18-L57; Cad/18-L22; CiudR/18-L27 | M159L | 13,524 |  | INS15 ATCATCATCATCATC | 6 | INS5 D164 |
| Cord/18-L17 | M160L | 12,662 | T | Y (C or T) |  | Possible change Q239-H |
| Bal/18-3532_5; Tol/18-1981_4; Cac/19-2681_8; Avil/19-2741_10; Avil/19-2815_1; Pal/19-2865_4 | M160L | 12,969 | A | T |  | Truncates protein Δ137-351 (end) |
| All samples | M160L | 12,912 | S (G or C) | C |  | CGA / CCA = R / P fixed to CGA=R156 |
| Vall/19-2585_2 | M160L | 13,020 | C | T |  | R120-K |
| Vall/19-2585_2 | M160L | 13,022 | C | T |  | No change |
| Vall/19-2585_2 | M160L | 13,031 | A | C |  | No change |
| Bal/18-3532_5; Tol/18-1981_4 | M160L | 13,044 | T | K (G or T) |  | Possible change H112-P |
| All samples | M160L | 13,083 | M (A or C) | C |  | ATK fixed to ATG=M98 |
| All samples | M160L | 13,085 | M (A or C) | C |  | GGG /GTG fixed to GGG=G99 |
| Vall/19-2614_1; Alb/19-1326_4 | M160L | 13,134 | G | R (A or G) |  | S82 or F82 |
| Cord/23-25IT_14 | M160L | 13,296 | - | T INS |  | Truncates protein Δ31-351(end) |
| Cord/20-ha33 | M160L | 13,365 | - | T INS |  | Truncates protein Δ8-351(end) |

^a^ Genome position refers to ha-MYXV reference genome Genbank accession number MK340973.

^b^ Amino acid position in the predicted ORF.
