# Supplementary figures and images for: Enrichment and Reduction of Microsatellite Regions in the Myxoma Virus Genome Following Species Jump to the Iberian Hare (Lepus granatensis)
Source: Transbound Emerg Dis. 2026 Apr 18;2026:3847131. doi: 10.1155/tbed/3847131 (PMC13091234; doi:10.1155/tbed/3847131)

## Slide 1
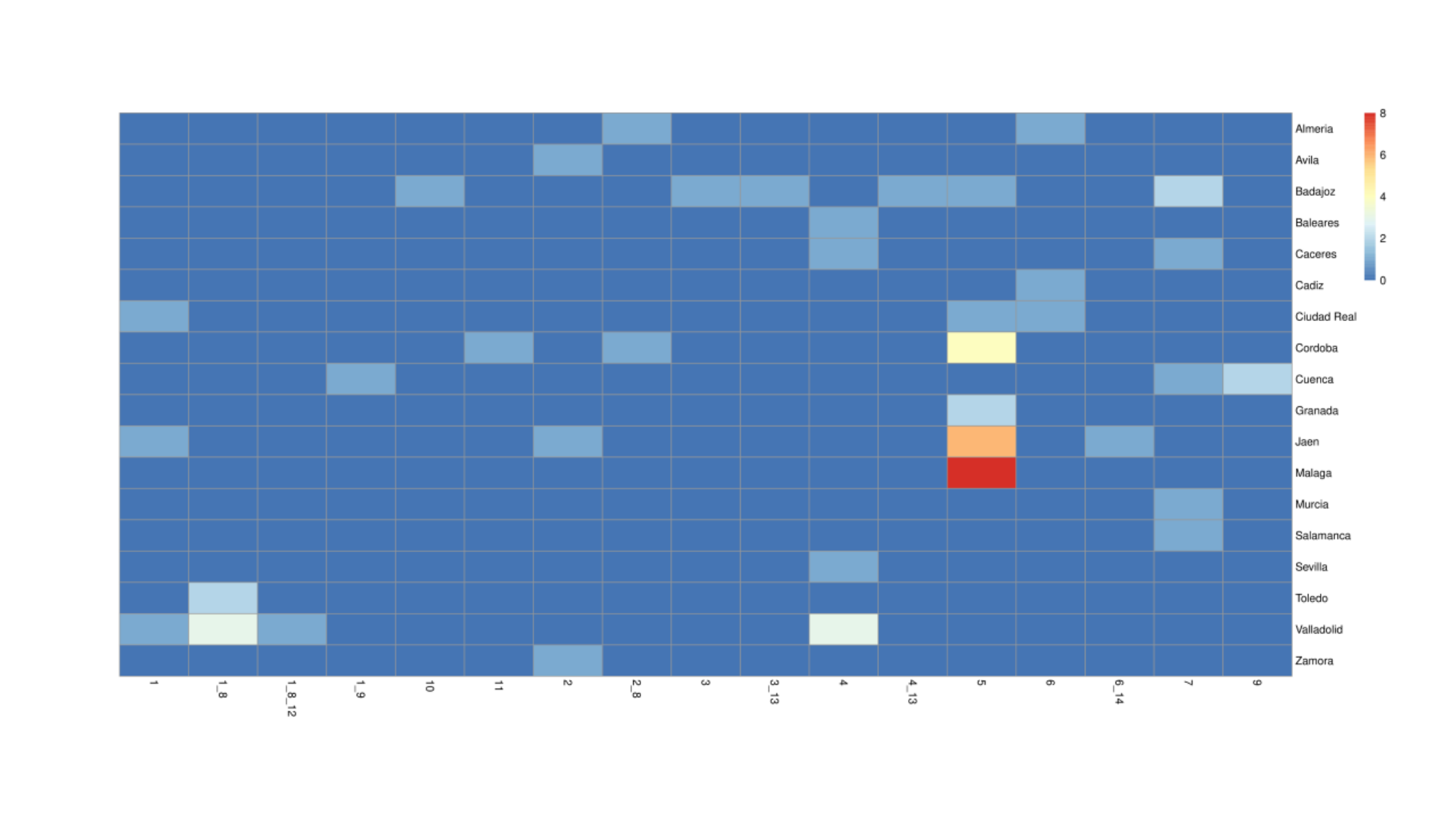

Supplement: Supplementary file 5 — Supporting Information 5 Figure S1: Heatmap showing the distribution of detected haplotypes across provinces based on SSR 159 sequence analysis. Each mutation type was designated a number, and combinations of mutation types present in samples were used to define individual haplotypes. Haplotype numerical codes are defined in Supporting Table 2 and are shown on the X‐axis, province names are shown on the Y‐axis. [file TBED-2026-3847131-s005.pptx]
